# POCUS in perioperative medicine: a North American perspective

**DOI:** 10.1186/s13089-017-0075-y

**Published:** 2017-10-09

**Authors:** Lorenzo De Marchi, Massimiliano Meineri

**Affiliations:** 1 Department of Anesthesia, Georgetown University, MedStar Georgetown University Hospital, 3800 Reservoir Road NW, CCC Building, Lower Level, Washington, DC USA; 2 Department of Anesthesia and Pain Management, Toronto General Hospital, University Health Network, University of Toronto, 200 Elizabeth Street EN 3-400, Toronto, ON M5G 2C4 Canada

**Keywords:** Point-of-care ultrasound, Perioperative medicine, Guidelines, Hand-held ultrasound devices

## Abstract

Ultrasound (US) performed at the point of care has found fertile ground in perioperative medicine. In the hands of anesthesiologists, transesophageal echocardiography (TEE) has become established as a powerful diagnostic and monitoring tool in the perioperative care of cardiac and non-cardiac patients. A number of point-of-care US (POCUS) applications are relevant to perioperative care, including airway, cardiac, lung and gastric US. Although guidelines exist to define the scope of practice for basic and advanced TEE, there remains a lack of such guidelines for perioperative point-of-care ultrasound (POCUS), despite a number of recent calls for action in the academic anesthesia community. POCUS training has been integrated into anesthesia residency curricula in Canada and the United States of America (USA). However, a nation-wide curriculum is still lacking. Many limitations to the development of perioperative POCUS curricula exist, including the need to define the scope of practice and design integrated longitudinal learning approaches. The main anesthesiologist societies in both the USA and Canada are promoting the development of guidelines and have introduced POCUS courses into their national conferences. Although bedside US imaging has been integrated into the curricula of many medical schools in North America, the need for specific national guidelines for the training and practice of POCUS in the perioperative setting by anesthesiologists is crucial to the further development of POCUS in perioperative medicine.

## Introduction

Point-of-care ultrasound (POCUS) refers to the use of ultrasound (US) examinations at a patient’s bedside by a primary healthcare provider to answer a limited number of specific diagnostic questions, guide treatment and invasive procedures. In the past decade, the role of this technology during the perioperative period has become increasingly recognized.

The use of US has become an established standard of care in most operating rooms for several applications, such as regional anesthesia and central line insertion [1]. Concurrently, transesophageal echocardiography (TEE) performed by anesthesiologists has become an established practice in the cardiac operating room, supported by specific guidelines in both the United States of America (USA) and Canada [2, 3]. This has helped increase the availability of US systems in the operating room and develop specific postgraduate curricula to teach US-guided line insertion and confirmation of placement and regional blocks [4–6]. Additionally, hand-held systems have become increasingly available for many physicians and acute care teams [7, 8].

A number of specific applications of POCUS assist providers in responding to the challenges of perioperative anesthetic care. Focused cardiac US [9] has been used in the perioperative assessment of elective [10] and urgent [11] surgical cases and has significantly impacted patient management [12–14]. POCUS may also play key roles in the management of hemodynamic instability and cardiac arrest [15]. Lung US not only exhibits high accuracy in the differential diagnosis of hypoxia [16] but also has been successfully used intraoperatively to detect tracheal intubation [17], exclude main stem bronchial intubation and confirm lung isolation in thoracic surgery [18, 19]. Upper airway US has been demonstrated to be more reliable than manual palpation in identifying the cricothyroid membrane [20] potentially reducing the risk of complications or failure in case of an emergency surgical airway.

Finally, while controversial, gastric US has become a unique tool in the hands of anesthesiologists for quantifying gastric contents and more objectively assessing aspiration risk [21]. However, this method is user dependent and subjective and is not standard of care in deciding if the patient is properly NPO.

More recently, specific perioperative scanning protocols have been proposed [22], and extensive reviews have advocated the need for anesthesiologists to perform perioperative POCUS [23]. Editorials in major anesthesia journals in the USA [24–26] and Canada [27] have clearly indicated that POCUS is the tool of the future in perioperative medicine. In 2011, an editorial by Johnson advocated for the development of POCUS into a standard of care for anesthesiologists [28]. Despite this editorial and other call for action, little progress has been made, and there exists no clear track toward a unified POCUS certification for anesthesiologists in North America.

In a recent survey of members of the Society of Cardiovascular Anesthesiologists, among 349 cardiac anesthesiologists who responded, most of whom were practicing in the USA, fewer than half had integrated focused cardiac US into their daily practice, and only 40% felt comfortable teaching this technique [29].

Although comprehensive [30] guidelines for the practice of POCUS are well established for emergency medicine and critical care in both the USA [30] and Canada [31], they are not immediately transferable to perioperative care, given the different scopes of practice. Another potential limitation restricting increases in the frequency with which POCUS is utilized is the relatively small number of anesthesiologists practicing in critical care in North America.

## Where are we?

The American Society of Echocardiography and the Society of Cardiovascular Anesthesiologists have established guidelines regarding basic perioperative TEE that define the use of TEE as a monitoring tool outside of the cardiac operating room. National Board of Echocardiography certification in basic perioperative TEE is available after the completion of a written exam (for Testamur status) and the submission of a complete case log (for Diplomate status). Basic TEE certification is not supported by updated Canadian guidelines and is limited to the intraoperative setting. Training in basic perioperative TEE nonetheless requires a significant time commitment and supervision.

Basic TEE is an ideal tool for perioperative hemodynamic monitoring, assessments of volume status, guidance for fluid administration, and rescue in cases involving hemodynamic instability [32]. The integration of basic TEE training into the residency curriculum [33] should be considered to offer the full potential of this tool to the next generation of providers [23, 34].

Similarity to focused TTE [9], a more simplified “focused” TEE limited to five views with the objective of answering dichotomous questions has been successfully used in emergency medicine [35] and critical care [36]. However, the intraoperative use of this technique has not been described to date.

The feasibility and effectiveness of basic focused TTE in Canadian anesthesia residency programs were described more than 5 years ago [4]. Research has demonstrated that in the USA, a comprehensive perioperative US curriculum significantly impacted anesthesia residents’ clinical assessment skills [34]. However, focused cardiac US teaching was found to be uncommon in anesthesia residency programs in the USA [37, 38], and a recent survey indicated that only 8 of 17 anesthesia residency programs in Canada offered any form of POCUS training [38]. In contrast, over 90% of cardiovascular anesthesiologists surveyed in the USA responded that focused cardiac US should be integrated into every anesthesia residency curriculum [37].

## What is next?

A structured pathway for developing postgraduate curricula has been proposed for critical care anesthesiologists [39] and for specific perioperative POCUS applications [40, 41].

POCUS curriculum development for anesthesia residency programs must be considered in the context of the new framework of competency by design [42]. The current Canadian model implies that all anesthesia training is built to be tested against all applicable CanMEDS roles. POCUS appears to be relevant to all seven of these roles (medical expert, professional, communicator, collaborator, leader, scholar and professional). From this perspective, POCUS would not be regarded as a separate technical skill but would instead be integrated into daily clinical practice. This development would require a number of steps, including defining the scope of practice for perioperative POCUS and designing a longitudinal curriculum that accounts for current evidence regarding training duration and structure [4, 43], simulator use, number of scans necessary to achieve proficiency, [9, 44–46] assessments of competence [47] and skill retention [48, 49]. National or international expert consensus may be required in areas for which no supporting evidence is available.

A separate challenge is to train the trainers; this challenge will lead to the creation of a certification pathway. Definitions of learning requirements will have to consider prior US experience hands-on learning and portfolio building will be limited by access to supervision and a lack of dedicated scanning time [50].

The introduction of integrated US teaching into medical school curricula is becoming standard across North America, and current graduates are therefore being trained to integrate US into clinical decision making. Residency curricula have a mandate to build on this foundation, and practicing anesthesiologists will need to prepare themselves for new teaching challenges.

Anesthesiologists are the main players in the perioperative care of surgical patients, from preoperative assessment to post-operative pain management. Therefore, they are the natural leaders in the context of the newly proposed perioperative surgical home model. With this prospect of more comprehensive longitudinal patient management, POCUS will find a fertile field of applications, given its proven roles in facilitating problem solving [51] and ensuring patient safety [34] during the pre-, intra- and post-operative periods.

POCUS requires adequate privileges, credentialing and oversight for quality assurance. At present, privileges to perform POCUS are currently granted by individual departments. There is no exemption for POCUS; this status may be problematic for medico-legal and remuneration purposes. In Canada and the USA, local regulations require electronic image storage and written reports for billing purposes. The availability of pictures archiving and communication systems (PACS) or web-based storage systems represents a significant concern, especially for community hospitals. Given the low remuneration for POCUS exams in both Canada and the USA, it is difficult to build a business model to support the practice of these examinations and justify the setup and maintenance costs of an image storage infrastructure. However, this challenge is a major limitation with respect to quality assurance and control.

## The future

An increasing number of anesthesia residency programs are integrating a mandatory POCUS curriculum. This development is echoed by increased interest in POCUS training targeted to practicing anesthesiologists. Nonetheless, guidelines are lacking.

As a start, in 2016, the Canadian Anesthesiologist’s Society formed a task force to develop a perioperative POCUS consensus statement that reflected the views of POCUS experts in all academic centers across the country. The first nation-wide, full-day perioperative POCUS course was held in Vancouver in June 2016. This course addressed all perioperative POCUS applications identified by the Perioperative POCUS Consensus Group.

The Canadian Emergency Ultrasound Society (CEUS) also developed the Emergency Department Echo Course in 2001, which became the standard POCUS certification course for emergency physicians in Canada. CEUS rebranded itself as the Canadian Point of Care Ultrasound Society (CPoCUS) (https://www.cpocus.ca) in 2016, and based on the strength of its certifying platform, offers certifications to anesthesiologists by combining different modules to fit applications of interest during the perioperative period.

In contrast, no major anesthesia society in the USA has embarked on the task of defining the scope of practice for POCUS or a path towards POCUS certification.

A special POCUS interest group was established last year within the American Society of Regional Anesthesia (ASRA).

However, the 2-day workshop on POCUS in anesthesia organized for 2017 [52] specially focused on focus assessed transthoracic echocardiography (FATE) certification and did not include all of the aforementioned components.

Thus far, the only other attempt to create a comprehensive POCUS certification has been offered by the Alliance for Physician Certification & Advancement (APCA) [53], a physician-centric council spun out of the American Registry for Diagnostic Medical Sonography (ARDMS).

The APCA is currently working to provide, a POCUS Fundamental Certificate covering basic knowledge regarding US. This certificate will be followed by a series of POCUS Clinical Certificates (cardiac, GI, lung, abdominal trauma, and GU certificates, among others) [53].

The acquisition of a predetermined number of POCUS Clinical Certificates will earn the provider a certificate in POCUS specific to a determined specialty.

The first available certification will be in emergency medicine. Additional specialty-dedicated certifications, including a certification in anesthesia, are planned for development in the future.

Even if the APCA certification system does not address the extension of training necessary to reach competency in POCUS, it may nonetheless represent a useful practical tool to evaluate the results of an appropriately formulated learning curriculum. Although a minimum number of scans will likely continue to be required, the creation of a curriculum based on a competency-based education model that will be more focused on the achievement of certain milestones than a predetermined number of exams will help to finally achieve this goal.

It is hoped that the increased interest in POCUS that has recently been expressed in numerous anesthesia publications will promote a movement that will lead to the formulation of a clear curriculum and guidelines by our professional societies.

To answer to the “call to action” from Mahmood et al. in last year’s June issue of *Anesthesia & Analgesia* [16], the present generation of anesthesiologists must have a well-defined and structured learning path to be able to teach subsequent generations.

## Abbreviations

POCUS: point-of-care ultrasound
US: ultrasound
TEE: transesophageal echocardiography
